# Calcium modifying dedicated balloons: a contemporary review

**DOI:** 10.3389/fcvm.2026.1700877

**Published:** 2026-04-10

**Authors:** Giuseppe Colletti, Zoltan Ruzsa, Olivier Gach, Adrien Jossart, Laura Peter, Agostino Spanò, Nino Cocco, Marouane Boukhris, Claudiu Ungureanu

**Affiliations:** 1 Department of Cardiology, Clinique St. Joseph—Groupe Vivalia, Arlon, Belgium; 2 Internal Medicine Department, Cardiology Center, University of Szeged, Szeged, Hungary; 3 Cardiovascular Center, Semmelweis University, Budapest, Hungary; 4 Department of Cardiology, CHC MontLégia, Liège, Belgium; 5 Department of Cardiology, Jolimont Hospital—CHU Helora, La Louvière, Belgium; 6 Department of Cardiovascular Sciences, Campus Bio-medico University Hospital, Rome, Italy; 7 Department of Cardiology, CHU Saint Etienne, Saint-Étienne, France

**Keywords:** calcified coronary lesion, cutting balloon, intravascular imaging, OPN NC balloon, percutaneous coronary intervention, scoring balloon, coronary artery disease

## Abstract

**Background:**

Calcified coronary lesions are a major challenge in percutaneous coronary intervention (PCI), frequently leading to stent underexpansion, restenosis, and adverse events. While multiple technologies exist for plaque modification, there remains a critical need for clear guidance on the optimal selection and use of dedicated balloon-based devices.

**Summary:**

This review summarizes current evidence on scoring balloons, cutting balloons, and ultra-high-pressure (OPN NC) balloons and aims to define their specific role and indications in the treatment of calcified coronary disease. Particular attention is given to how lesion morphology, calcium characteristics, and procedural context influence device selection. Scoring balloons use external nitinol elements to create controlled focal stress, particularly effective in eccentric calcium. Cutting balloons incorporate microblades mounted on the balloon surface, allowing intimal incisions and fracture of concentric and mixed calcification or fibrotic tissue; repeated sequential inflations, as proposed in the RODIN-CUT technique, may enhance calcium disruption in a dose-dependent manner. OPN NC balloons, capable of inflations up to 35–40 atm, provide an effective last-resort option in undilatable lesions. Across devices, intravascular imaging plays a pivotal role in characterizing calcium morphology, guiding device selection and sizing, and confirming plaque modification.

**Conclusions:**

Calcium-modifying balloons provide predictable and versatile strategies for treating calcified coronary disease suitable both as a first-line option and as a complementary tool alongside intravascular lithotripsy or atherectomy. Device performance is tightly linked to lesion selection, procedural technique, and intravascular imaging guidance. An individualized, integrated, and morphology-tailored multimodality approach remains the most pragmatic strategy.

## Highlights

- Calcified coronary lesions remain a major challenge in PCI, strongly associated with stent underexpansion and adverse outcomes.
- Scoring balloons are mainly effective on superficial calcified lesions; cutting balloons are best suited for fibrocalcific disease; OPN NC balloons are a valid alternative to intravascular lithotripsy in concentric thick calcified lesions.
- Intravascular imaging (IVUS and OCT) is essential for lesion characterization, device sizing, and confirmation of adequate plaque modification.
- The RODIN-CUT technique suggests a dose-dependent effect of repeated cutting balloon inflations, facilitated by sequential IVUS assessment.
- A multimodality approach integrating balloons with IVL or atherectomy provides the best strategy for managing complex calcium.
- Future directions include randomized evidence comparing balloon-based devices, as well as hybrid innovations combining cutting or scoring elements with ultra-high-pressure technology.

## Introduction

Coronary artery calcification remains one of the most challenging obstacles in contemporary percutaneous coronary intervention (PCI). Calcified plaques are encountered in up to 30% of cases, and their prevalence increases with patient age, diabetes mellitus, chronic kidney disease, and complex coronary anatomy (1, 2). The presence of calcium has important clinical implications, as it can hinder balloon expansion, limit stent delivery, and ultimately result in incomplete stent expansion and apposition. In turn, this is associated with an increased risk of in-stent restenosis, stent thrombosis, and adverse clinical outcomes (1–4). Another concern in the management of heavily calcified lesions is the increased risk of procedural complications, including vessel dissection, perforation, and slow or no reflow (5).

The clinical challenges posed by coronary calcification are strongly influenced by the morphology and distribution of calcium. Lesions may display concentric calcium, eccentric plaque, or focal nodular deposits protruding into the lumen. Each of these patterns creates distinct barriers to optimal lesion preparation and requires a tailored strategy. The increasing use of intravascular imaging has highlighted the variability of calcium morphology and the importance of adapting PCI strategies accordingly.

A broad armamentarium of calcium modification tools is now available, including atherectomy devices (rotational, orbital, laser), intravascular lithotripsy (IVL), and dedicated specialty balloons (6, 7). Atherectomy devices are effective but require significant operator expertise and carry a higher risk of complications such as perforation or slow flow (8). IVL has recently emerged as a promising option, using acoustic shockwaves to fracture calcium (9). However, IVL balloons still require lesion cross ability, and their high cost limits widespread use. Furthermore, IVL is to be considered as a whole different technique rather than a simple specialty balloon.

While these modalities have significantly improved the ability to modify calcium, they also introduce new challenges. Their use can increase procedural complexity, lengthen intervention time and carry specific procedural risks and, especially when combined, escalate the total cost of PCI.

Dedicated balloon-based devices—including scoring, cutting, and ultra-high-pressure (OPN NC) balloons—represent versatile, widely available, and relatively easy-to-use options (10). Importantly, these tools are particularly useful in balloon-crossable lesions, where they can effectively modify calcium and optimize vessel preparation prior to stenting. While these devices share a common purpose, their design and mechanisms of action differ substantially, influencing their impact on the calcified plaque and hence their clinical applications.

Importantly, the abundance of available devices has not been matched by clear, reproducible strategies to guide their use. Operators are frequently faced with the dilemma of how to select among multiple tools—and more critically, how and when to use them, in what order, and with what technical protocol. Intravascular imaging has helped to better characterize calcium morphology (11–16), but standardized pathways for matching lesion type to device and technique are still lacking. As a result, the treatment of calcified lesions often varies considerably between operators and institutions, leading to inhomogeneity in outcomes and cost-effectiveness.

Decision-making algorithms for the treatment of coronary calcium using intravascular imaging have already been proposed (6, 7). The purpose of this review is therefore not merely to summarize the technical features of calcium-modifying balloons (MB), but to provide a clinical and procedural framework for their use. We aim to define their place within the broader landscape of calcium treatment, based on lesion morphology, imaging guidance, and procedural context. Particular attention is given to how these devices are used—the number of inflations, balloon sizing, inflation pressure, and sequence—which are often as important as the device itself. In doing so, this article proposes a pragmatic, multimodal strategy for treating calcified coronary disease that balances efficacy, safety, and resource use.

## Scoring balloons

Scoring balloons were the first dedicated balloon devices designed specifically to enhance lesion preparation in calcified coronary plaques.

### Mechanism of action and design

Their fundamental concept is the incorporation of a scoring element—commonly a nitinol cage or spiral wires—wrapped around a semi-compliant balloon. Upon inflation, these scoring elements apply localized force to the arterial wall, resulting in controlled intimal scoring and fracturing of superficial calcium, while minimizing uncontrolled dissections compared to plain balloon angioplasty (17).

The mechanical action of scoring balloons is based on the principle of stress concentration. By channeling balloon pressure through scoring elements, high focal forces are applied at the contact points between the nitinol struts and the plaque. This creates controlled scoring lines, allowing the balloon to expand the lesion at lower pressures, thereby reducing the risk of vessel trauma (18, 19). Unlike cutting balloons, scoring balloons do not use microblades but instead rely on these circumferential or helical scoring wires to evenly distribute force.

**Figure 1:**
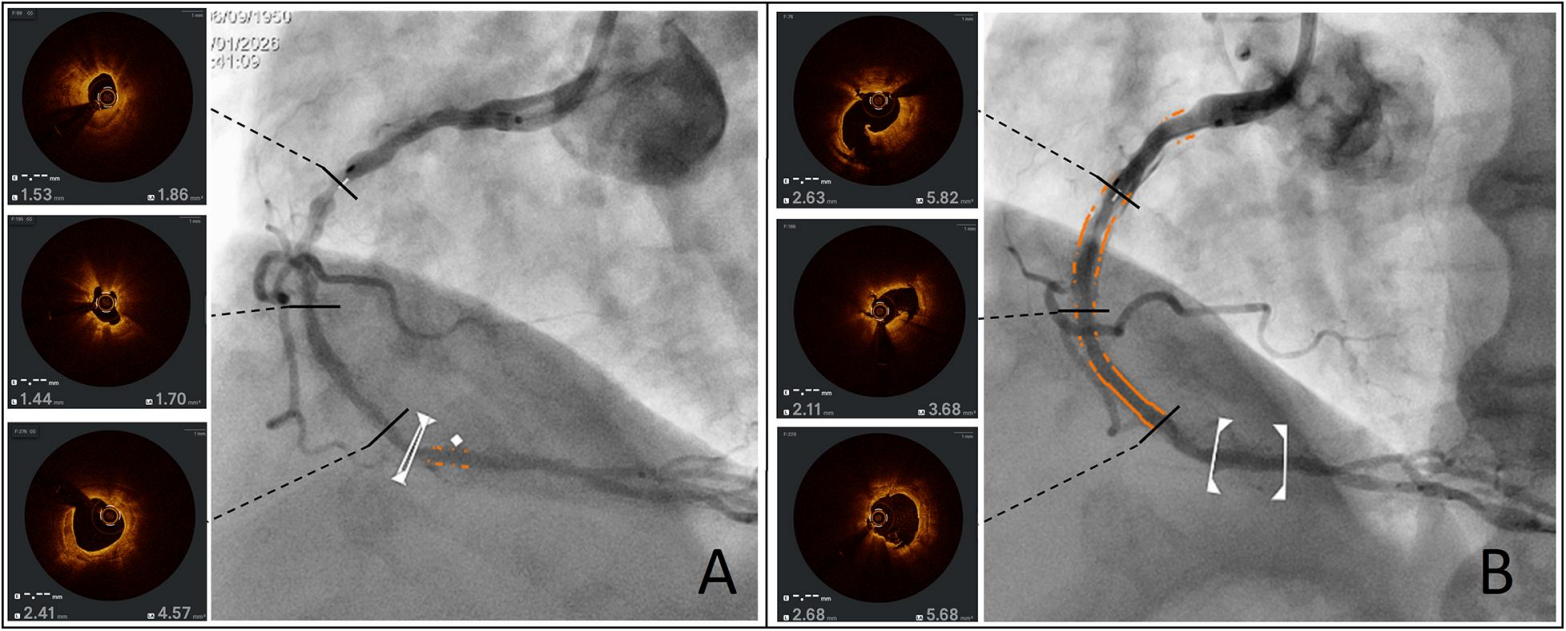
*Effects of a scoring balloon on severely calcified disease.* Panel **(A)** shows the OCT pullback of a severely calcified right coronary artery at baseline while Panel **(B)** shows how the same segments have been modified after lesion preparation with a 3 mm Angiosculpt™ scoring balloon with visible fractures and significant luminal gain.

Figure 1 depicts a severely diseased right coronary artery presenting severe calcifications showing the optimal coherence tomography (OCT) at baseline (Panel A) and after lesion preparation (Panel B) with a 3 mm Angiosculpt™ scoring balloon (Philips, Amsterdam, The Netherlands).

### Clinical evidence

The AngioSculpt™ scoring balloon was one of the first widely used scoring devices, and OCT imaging studies have demonstrated its ability to induce visible calcium fractures, improving vessel compliance and luminal gain (18). Similarly, the NaviScore™ scoring balloon system (Terumo Corporation, Tokyo, Japan) has shown feasibility and safety in routine practice, with registry data suggesting high device success and low complication rates (20). More recently, the Aperta NSE™ balloon (Nipro Corporation, Osaka, Japan) has attracted attention for its unique design with three scoring wires integrated in a NC balloon, offering enhanced pushability and trackability (21). Meanwhile the recent Wedge NC randomized trial has assessed the safety and efficacy of this novel scoring balloon against conventional balloons in calcified coronary disease (22).

Scoring balloons have also demonstrated a valuable role in lesion preparation for drug-coated balloon (DCB) angioplasty, as shown in studies where their use facilitated adequate vessel compliance and reduced recoil, thereby improving drug delivery and clinical outcomes (17).

Evidence from randomized comparisons between modified balloons and atherectomy provides further perspective. The PREPARE-CALC randomized trial reported higher strategy success with rotational atherectomy compared with modified balloons, though both approaches achieved similar acute lumen gain and comparable 9-month outcomes when used with new-generation DES (23).

An OCT sub-study of this trial demonstrated that stent expansion, eccentricity, and asymmetry were similar between groups, with calcium morphology—particularly plaque length and thickness—emerging as the main predictors of expansion (24).

Long-term follow-up at two years confirmed no significant differences in target vessel failure or MACE, though rotational atherectomy appeared more favorable in longer lesions, while modified balloons were equally effective in short lesions (25).

At 5 years follow-up comparable rates of TVF were reported. However, a significant reduction of TLR was observed after RA showing potential clinical advantages of RA over MB during long-term follow-up (26).

Meta-analyses provide complementary insights. A pooled analysis of >2,700 patients showed that cutting and scoring balloons reduced target lesion revascularization compared with conventional balloons, without excess complications (27).

Conversely, a meta-analysis restricted to randomized trials found no consistent improvement in acute or imaging outcomes over standard balloons (28).

### Practical considerations

When using scoring balloons, correct sizing is critical. They should generally be sized 1:1 relative to the reference vessel diameter, ensuring adequate plaque modification without excessive oversizing. Recommended inflation pressures typically range between 12 and 20 atm, with gradual stepwise inflation to avoid vessel trauma. Their strengths include good deliverability, controlled plaque modification, and relative safety in both calcified and fibrotic lesions.

Scoring balloons are particularly useful mainly in concentric and eccentric calcification, where focal scoring lines facilitate subsequent stent expansion, and less in fibrotic lesions, where their role consists more on reducing balloon's slippage. Their main limitations include somewhat reduced efficacy in deep calcifications or nodules, where deeper plaque disruption may be required. In such cases, additional devices such as cutting balloons, IVL, or OPN balloons may be necessary.

A special technique, namely the bending technique, has been described to increase crossability when using the Aperta NSE™ scoring balloon (29). The special design of this balloon allows in fact to modify and retain the balloon shape potentially improving its crossability in tortuous anatomies.

## Cutting balloons

Cutting balloons were developed to overcome the limitations of conventional angioplasty in resistant fibrocalcific lesions. These devices feature microsurgical blades (atherotomes) mounted longitudinally on the surface of a non-compliant balloon. Upon inflation, the blades make controlled incisions in the intima and plaque that might extend to the deeper media, enabling lesion expansion at lower pressures and with improved predictability compared to plain balloon angioplasty (30).

### Mechanism of action and device design

The blades of cutting balloons concentrate pressure into sharp focal points, producing controlled intimal dissections and facilitating plaque fracture. This allows expansion at lower balloon pressures, reducing uncontrolled dissections and vessel trauma. Unlike scoring balloons, which rely on nitinol wires to create scoring lines, cutting balloons actively cut into the plaque, generating deeper and more defined incisions (19, 31–33).

The most commonly used modern cutting balloon is the Wolverine™ cutting balloon (Boston Scientific, Marlborough, MA, USA), which has three or four microblades attached to a non-compliant balloon. Bench studies and finite element analyses have confirmed its ability to produce effective calcium fracture patterns while minimizing vessel overstretch (31–33).

**Figure 2:**
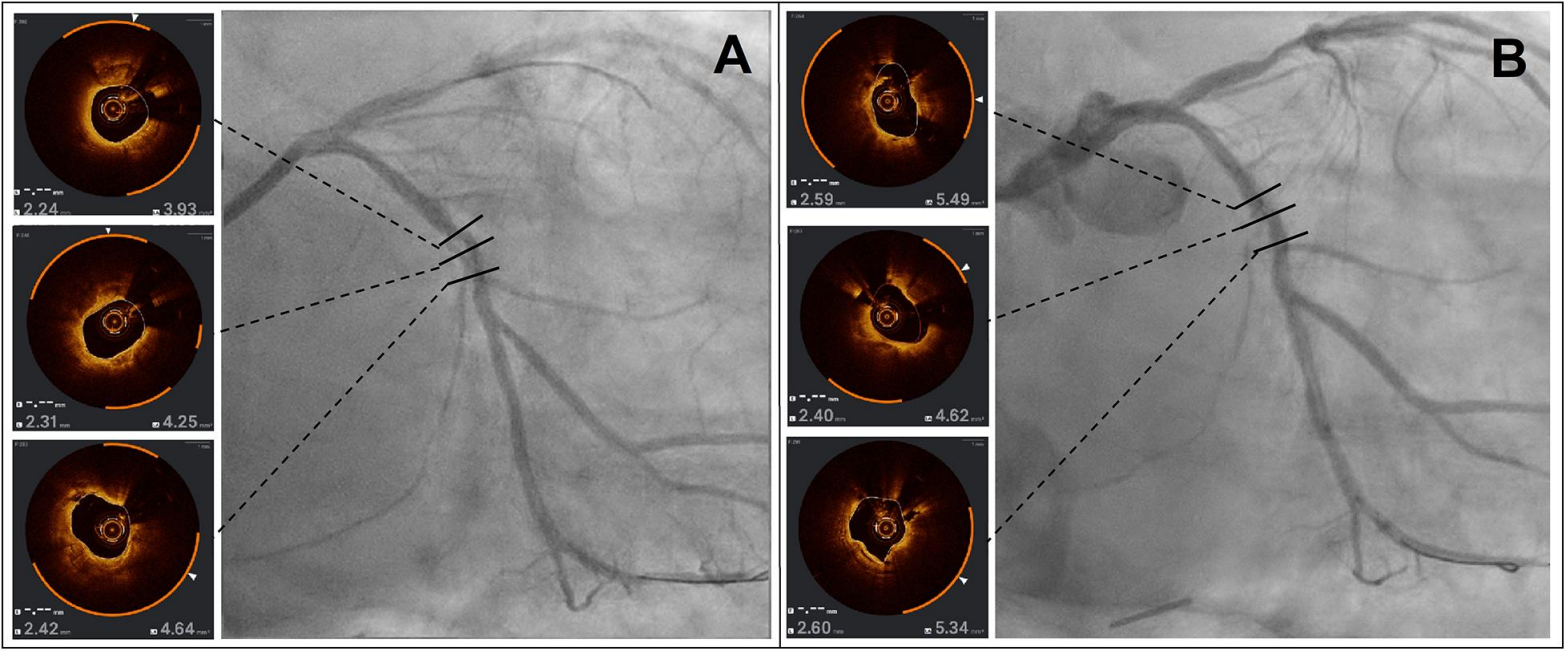
*Effects of a cutting balloon on severely calcified disease.* Panel **(A)** shows the OCT pullback of a severely calcified lesion over the Circumflex artery at baseline while Panel **(B)** shows how the same segments have been modified after lesion preparation with a 3 mm Wolverine™ cutting balloon with visible fractures and significant luminal gain.

Figure 2 depicts a severely calcified lesion on the Circumflex artery showing the OCT at baseline (Panel A) and after lesion preparation with a 3 mm Wolverine™ cutting balloon (Panel B).

### Clinical evidence

The role of cutting balloons in complex PCI has been evaluated in multiple randomized trials and registries. The Cutting Balloon Global Randomized Trial showed comparable restenosis rates to conventional angioplasty in *de novo* lesions, but highlighted reduced vessel recoil and predictable luminal gain (34). The RESCUT trial further confirmed their effectiveness in the treatment of in-stent restenosis, with improved acute luminal gain compared to conventional balloons (35).

More recently, the COPS-1 Trial demonstrated the utility of cutting balloons for lesion preparation prior to stent implantation, showing improved stent expansion and lower rates of stent underexpansion compared with non-modifying balloons (30).

Preliminary data from the Short-CUT trial reported that cutting balloon therapy is non-inferior to IVL in terms of final minimal stent area (31). However, among patients without rotational atherectomy pre-treatment, non-inferiority for minimal stent area was not met; this finding should be interpreted in the context of smaller vessel size in this subgroup compared with the IVL cohort, despite similar stent expansion criteria between groups, suggesting that cutting balloons may require combination calcium modification strategies in selected heavily calcified lesions.

Indeed, observational studies have shown benefits in combining cutting balloons with other devices, such as rotational atherectomy (the ROTA-CUT strategy), suggesting a potential impact on outcomes (36–38). Although a recent meta-analysis has confirmed the safety of this approach showing no impact on major outcomes when compared to atherectomy followed by plain NC balloon dilatation (39), while the first conducted randomized trial failed to show a significant difference in MSA between the 2 approaches (40).

The novel concept of repeated sequential cutting balloon inflations (RODIN-CUT technique) has recently emerged as a potential way to enhance the effectiveness of this device (41). By alternating cutting balloon inflations with intravascular ultrasound (IVUS) evaluation—both performed without removing either system from the 7Fr guide catheter—operators observed progressive plaque disruption and calcium fragmentation. In fact, each cutting balloon inflation modifies both the plaque and vessel architecture. This, together with the different balloon unfolding and folding patterns at each inflation, ultimately results in a change related to the contact points of the microblades with the surrounding structures. A dose-dependent effect is hence likely present, where multiple inflations may incrementally enhance calcium modification. The ongoing retrospective RODIN-CUT registry is expected to provide further insights into the clinical utility of this approach.

Of note, case reports have described blade fracture during cutting balloon inflations in severely calcified lesions, highlighting both the effectiveness and mechanical limitations of this technology (42). These findings emphasize the need for careful lesion selection and proper balloon sizing.

Finally, the deeper intimal dissections created by cutting balloons might increase the efficacy of drug coating balloon angioplasty for *de novo* lesions, as some studies have shown them to be associated with a significant improvement in late lumen enlargement (43, 44).

### Practical considerations

The traditional use of cutting balloons consists of sizing it generally following a 1:1 ratio with the reference vessel diameter, though in very calcified or tortuous segments some operators prefer undersizing slightly to reduce perforation risk. Inflation pressures typically range from 6 to 12 atm, substantially lower than those required for standard non-compliant balloons, as effective cutting is achieved at modest pressures. The inflation pressure anyhow should be adapted depending on the sizing of the balloon related to the vessel diameter. Higher pressures could be applied for undersized balloons.

Cutting balloons are particularly effective in fibrotic, concentric and eccentric calcified lesions, as well as, although less significantly, in nodular calcium. They are also a valuable adjunct in in-stent restenosis, reducing elastic recoil and improving luminal gain. However, in very severe concentric or long calcification, cutting balloons may have limited efficacy unless combined with other strategies.

While more data are still needed, the application of multiple inflations, as in the RODIN-CUT approach, is advised as it might result in better calcium fragmentation although a recently published case series suggests an increased risk of occurring blades fracture (42).

Their advantages include predictable plaque modification, facilitation of stent expansion, and reduced need for extreme balloon pressures.

Their disadvantages include reduced deliverability in tortuous anatomy, risk of blade fracture, and risk of vessel perforation if oversized or inflated aggressively.

## Ultra-high pressure balloons (OPN NC)

Ultra-high-pressure (UHP) balloons, most notably the OPN NC™ balloon (SIS Medical AG, Winterthur, Switzerland), are specifically designed to tackle the most resistant calcified lesions.

### Mechanism of action and device design

The OPN NC™ features a dual-layer balloon design: an inner balloon that maintains shape and an outer balloon that distributes force evenly, allowing inflation up to 40 atm or more without risk of rupture (45).

This unique engineering enables the balloon to deliver extremely high focal forces against calcified plaques, producing controlled cracking of calcium and expansion of lesions otherwise resistant to conventional or specialty balloons. Unlike cutting or scoring technologies, OPN NC™ balloons rely purely on mechanical pressure, but the dual-layer design minimizes the risk of uncontrolled dissections or balloon burst.

**Figure 3:**
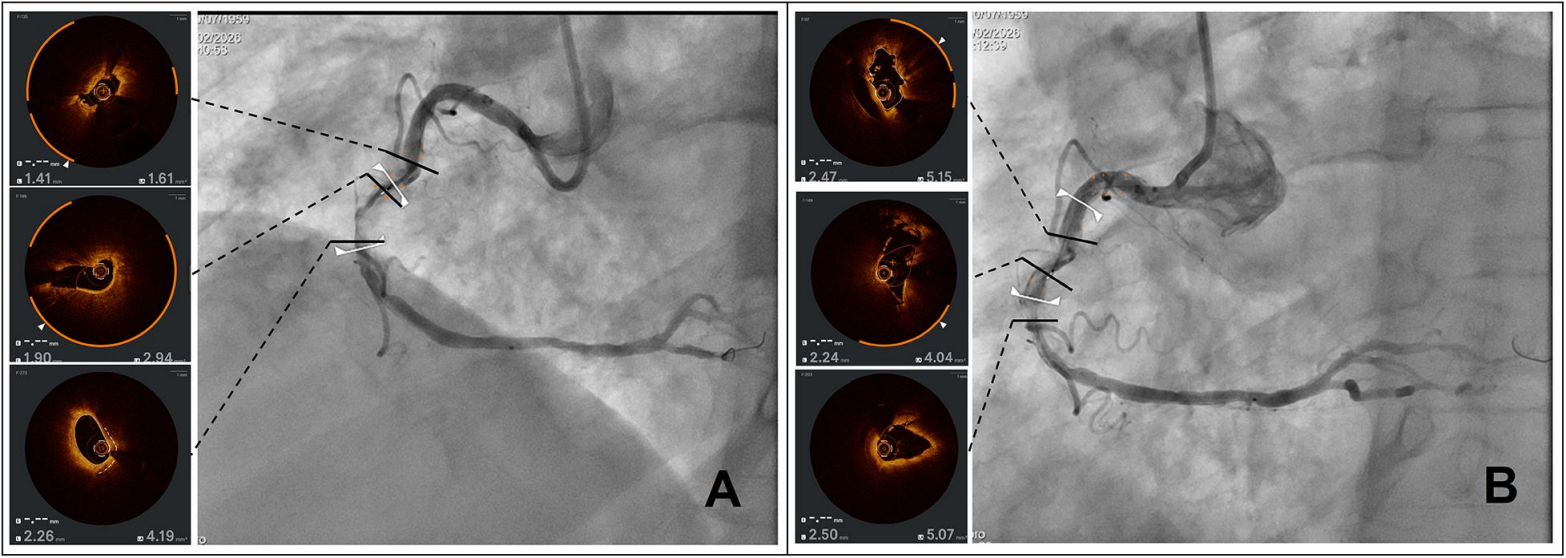
*Effects of an ultra-high pressure NC balloon on severely calcified disease.* Panel **(A)** shows the OCT pullback of a severely calcified right coronary artery at baseline while Panel **(B)** shows how the same segments have been modified after lesion preparation with a 3 mm OPN NC™ ultra high pressure balloon with visible fractures and significant luminal gain. Notably the OCT frame below shows an efficacy of the balloon in “tearing” fibrotic tissue.

Figure 3 depicts a severely diseased right coronary artery with severe calcifications showing the OCT at baseline (Panel A) and after lesion preparation with a 3 mm OPN NC™ balloon (Panel B).

### Clinical evidence

The safety and efficacy of ultra-high-pressure balloons have been documented in multiple registries and systematic reviews. Initial reports demonstrated their ability to achieve expansion in undilatable lesions, with very high procedural success rates and low rates of complications (46, 47).

The ISAR-CALC randomized trial directly compared OPN NC™ balloons to scoring balloons in heavily calcified lesions. OPN NC™ achieved superior acute luminal gain and stent expansion, with similar safety outcomes (48). A more recent systematic review and meta-analysis confirmed that OPN NC™ balloons provide high success rates in resistant lesions, though at the expense of longer procedural times and higher use of adjunctive devices (49). The same meta-analysis had confirmed the relatively safety of this device showing perforation rates below 1% and MACE incidence around 2%.

Registry data, including multicenter OCT-based studies, have illustrated how OPN NC™ balloons produce visible calcium fractures, especially in concentric thick calcifications (50).

Additional support comes from the pooled analysis of the PREPARE-CALC and ISAR-CALC randomized trials (*n* = 274), which showed that stent expansion was comparable among rotational atherectomy, modified balloons, and OPN NC™ balloons, but OPN NC™ balloons were associated with less stent eccentricity (51).

Moreover, preliminary data from the VICTORY trial, presented at the Transcatheter Cardiovascular Therapeutics (TCT) 2025 meeting, suggest that the OPN non-compliant balloon is non-inferior to intravascular lithotripsy (IVL) in terms of stent under-expansion (52).

Nonetheless, their position in the treatment algorithm remains debated. While highly effective, OPN NC™ balloons are typically reserved for balloon-crossable but undilatable lesions that fail to yield after non-compliant, scoring, or cutting balloons. Their role is therefore complementary rather than first-line, serving as a “last resort” before moving to atherectomy or IVL.

### Practical considerations

Slightly undersizing of OPN NC™ balloons of 0.5 mm compared to the reference vessel diameter is highly advised (47). The same principle applies whenever its use is intended to tackle stent underexpansion. Inflation should begin at 12–16 atm and be progressively increased in a controlled fashion, with careful monitoring, up to 40 atm as needed. Rapid full-pressure inflation should be avoided to minimize vessel injury.

These balloons are particularly indicated for severe concentric calcification where other devices fail to achieve expansion. They may also prove effective in fibrotic in-stent restenosis or situations where atherectomy is not feasible. Their main advantages are unmatched dilating force and ability to salvage undilatable lesions without requiring atherectomy. Their disadvantages include reduced trackability in tortuous anatomy, longer inflation times, and potential for vessel trauma if used aggressively.

The use of OPN NC™ balloons is associated to wire friction and potential wire entrapment and loss when retrieval is attempted. This largely depends on the guidewire's profile that is being used. As shown in a bench test study, Sion Blue™ guidewire (Asahi Intecc, Aichi, Japan) carries the higher risk of wire entrapment while on the opposite side BMW™ guidewire (Abbott Vascular, Santa Clara, CA, USA) carries the lowest (53).

Moreover, the use of an extra-support, non-hydrophilic guidewire such as the Grand Slam™ guidewire (Asahi Intecc, Aichi, Japan) may offer benefits in PCI procedures**.** Specifically, extra-support wires can enhance device deliverability by providing increased shaft support, which may reduce wire–balloon shaft interaction and facilitate crossing of complex or resistant lesions. In addition, the Grand Slam™ guidewire features a short distal coil segment with limited hydrophilic coating, potentially making it less susceptible to microstructural damage or deformation induced by extreme inflation pressures compared with wires with long hydrophilic segments.

However, evidence supporting these advantages remains limited and is largely derived from technical reports, case series, and mechanistic considerations rather than randomized data (53).

Alternatively, to further reduce the risk of wire loss or balloon entrapment, OPN NC balloons can be advanced over a second guidewire, providing additional support and stability during device delivery.

## The role of intravascular imaging

Optimal treatment of calcified coronary lesions increasingly relies on intravascular imaging modalities such as intravascular ultrasound (IVUS) and optical coherence tomography (OCT). These tools provide detailed insights into plaque morphology, distribution, and thickness, which are not adequately visualized by angiography alone (11).

### Imaging-based calcium assessment

Angiography underestimates the severity and distribution of coronary calcification. IVUS and OCT enable accurate quantification of calcium arc, thickness, and length, parameters strongly associated with procedural success and risk of stent underexpansion (12).

The IVUS-based calcium score developed by Zhang et al. identifies lesions at high risk for stent underexpansion when three criteria are met: 1. superficial calcium arc >270°, 2. calcium length >5 mm, and 3. vessel diameter <3.5 mm (13). Similarly, the OCT-based calcium score developed by Fujino et al. and recently revised, integrates arc, thickness, and length of calcium to predict optimal stent expansion. A calcium arc >270° with a length longer than 3 mm, calcium angle of 360° and thickness >0.3 mm are strongly predictive of inadequate expansion if plaque modification is not performed (14).

These imaging-based scores provide an objective framework to guide the selection of plaque modification techniques.

Moreover, AI-powered OCT software has been developed for automated calcium detection and quantification. By providing rapid and reproducible assessment of calcium morphology, these tools may complement established imaging-based scores and facilitate lesion preparation strategies, although further clinical validation is warranted (54).

### Morphological insights and device selection

Different calcium morphologies have distinct implications for treatment strategy:

Eccentric calcium: often more amenable to scoring balloons, which create linear fractures facilitating compliance.
Concentric calcium: frequently requires more aggressive modification with cutting balloons or even OPN balloons if concentric and thick.
Nodular calcium: represents the most challenging subset, often necessitating atherectomy or intravascular lithotripsy (IVL), but cutting balloons may be considered as adjunctive therapy.

OCT, with its high axial resolution, can directly visualize calcium fracture after balloon inflations, providing immediate feedback on treatment efficacy. IVUS, on the other hand, offers greater depth penetration and vessel sizing accuracy, which is critical for selecting balloon diameter (15, 16).

### Integrating imaging into procedural strategy

Imaging should not be seen as optional but as an integral part of contemporary PCI in calcified lesions. Pre-procedural IVUS or OCT defines the need for plaque modification, identifies optimal balloon size, and clarifies whether a lesion is balloon-crossable. Post-modification imaging confirms whether adequate plaque disruption has been achieved, or whether additional steps (repeat ballooning, higher pressure, or switching devices) are needed (15, 16).

In this sense, intravascular imaging had become a procedural partner in what may be considered a “live” active process, where device performance is continuously evaluated and optimized. Integrating imaging at multiple timepoints enhances safety, improves procedural efficiency, and maximizes the likelihood of adequate stent expansion.

The development of strategies such as the RODIN-CUT technique has been largely facilitated by intravascular imaging. By combining cutting balloon inflations with sequential IVUS assessments, operators observed incremental calcium fragmentation and changes in vessel architecture, supporting the concept of a dose-dependent effect of repeated cutting balloon use (41).

Thus, intravascular imaging not only informs the choice of scoring, cutting, or OPN balloons but also refines their application, ensuring effective yet safe lesion preparation.

## Upcoming alternatives

Among the upcoming alternative strategies, the LithiX™ Hertz Contact IVL system (Elixir Medical Corporation, Milpitas, CA, USA) has recently been introduced as a device incorporating multiple discrete metal hemispheres on a semi-compliant balloon. Through the generation of localized focal stress points, this system enables calcium fragmentation across a wide spectrum of moderate to severe calcified morphologies, while its design obviates the need for an external power source and may facilitate procedural workflow. However, clinical evidence supporting this technology remains limited. To date, the main source of data derives from the ongoing PINNACLE I trial, a prospective, multicenter, single-arm study including 60 patients (55). This investigation reported favorable safety outcomes and encouraging OCT findings in terms of calcium modification and subsequent stent expansion (56), suggesting the potential role of this novel system in the management of severely calcified coronary lesions.

Ongoing technological advances in this field are also expected to enable the development of coronary angioplasty balloons capable of sustaining ultrahigh inflation pressures and improved cross ability, thereby broadening future treatment options.

Additionally experimental concepts, such as balloons equipped with real-time intravascular imaging (e.g., IVUS/OCT) and fully-automated 3D OCT-derived simulations for treatment planning, represent promising avenues for the future, although these remain largely in preclinical or developmental stages.

## Discussion

The management of calcified coronary lesions remains one of the most challenging aspects of percutaneous coronary intervention (PCI). Residual calcium is strongly associated with stent underexpansion, restenosis, and stent thrombosis, making adequate lesion preparation a crucial determinant of long-term outcomes (4). Despite advances in drug-eluting stents and adjunctive pharmacology, optimal stent deployment cannot be achieved without first addressing the mechanical barrier of calcium (5).

Balloon-based devices play an essential role in this regard. Unlike atherectomy, which is generally reserved for balloon-uncrossable lesions, dedicated balloons are applicable in the broader group of balloon-crossable lesions. Scoring balloons, cutting balloons, and ultra-high-pressure (OPN NC™) balloons each provide unique advantages. When used judiciously, they allow controlled fracture of calcium, plaque modification, and more predictable stent expansion while maintaining procedural simplicity compared with atherectomy.

Recent innovations have emphasized how the performance of these devices can be optimized. The RODIN-CUT concept, developed through systematic integration of intravascular ultrasound (IVUS) after each inflation, has revealed that repeated cutting balloon inflations may have a dose-dependent effect on plaque modification (41). Each cycle of inflation and deflation may both progressively disrupt the vessel architecture and reorient the microblades, allowing them to engage different portions of the calcium arc. This sequential approach appears to enhance calcium fracture and vessel compliance beyond that achieved with a single inflation. The upcoming RODIN-CUT registry will provide data on the reproducibility, safety and efficacy of this strategy. At the same time, operators must remain aware of potential complications. Case reports have described blade fracture after repeated cutting balloon use, highlighting the need for careful device handling and attention to inflation pressures (42).

No single device can address all morphologies of coronary calcium. Instead, an integrative, multimodality approach is required. In practice, scoring balloons are frequently most effective for eccentric calcium or fibrotic lesions; cutting balloons perform well in concentric or mixed disease; intravascular lithotripsy (IVL) may be considered when deep calcium sheets or nodules limit vessel compliance; and OPN NC™ balloons serve as the last resort for undilatable lesions resistant to conventional techniques. Such stepwise escalation allows individualized therapy and avoids unnecessary reliance on any one modality.

**Figure 4:**
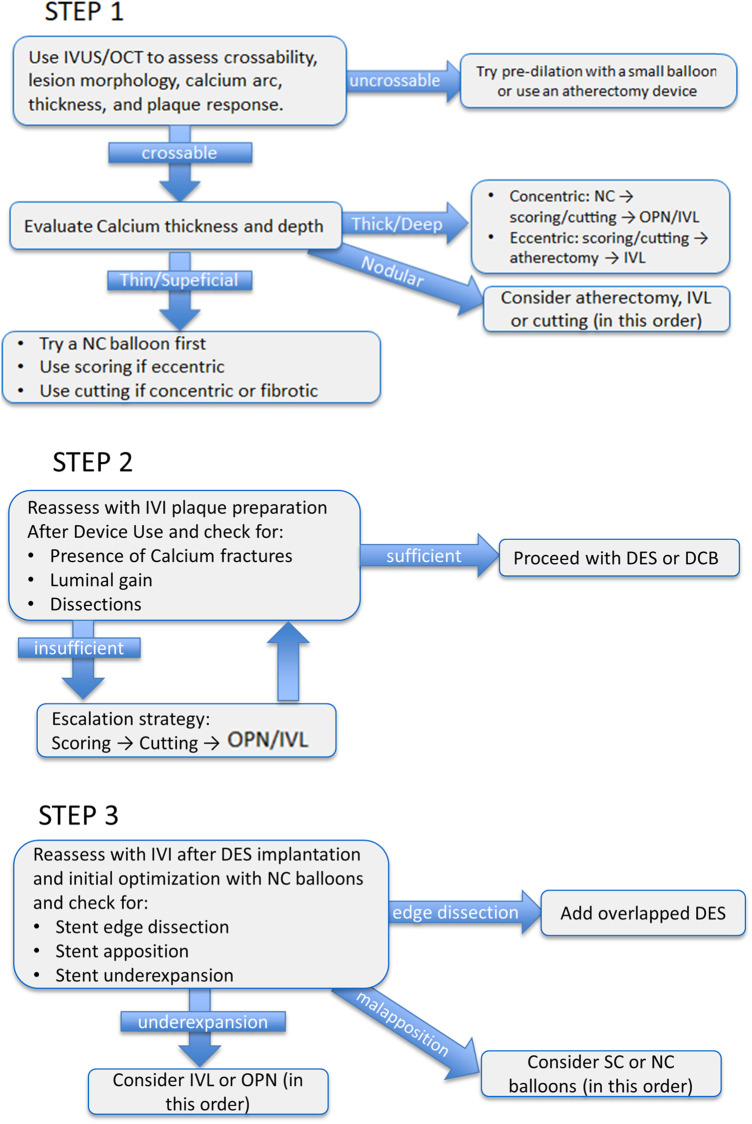
*Pragmatic algorithm for balloon-based calcium modification in percutaneous coronary intervention.* The algorithm integrates pre-procedural imaging with stepwise device selection and escalation. Step 1: intravascular imaging (IVUS/OCT) is used to check for lesion crossability and to characterize lesion morphology to help choosing for first-line treatment device. Step 2: intravascular imaging is used to evaluate plaque preparation after use of the selected device and to establish whether an escalation strategy should follow. Step 3: after stent deployment and initial optimization with conventional NC balloons final intravascular imaging is performed to evaluate stent expansion, apposition and edge dissections.

Table 1 provides a comparative summary of the main characteristics, strengths, limitations, and supporting evidence for the different dedicated calcium-modifying balloons, highlighting their complementary roles in contemporary PCI while in Figure 4 we propose a pragmatic algorithm that could guide on their use balancing efficacy, safety, and resources use.

**Table 1:** Key features of dedicated calcium-modifying balloons.

	Scoring balloons	Cutting Balloons	OPN NC (Ultra-high-pressure) Balloons
Mechanism of action	Semi-compliant balloon with external nitinol wires/cage; stress concentration causes controlled intimal scoring	Non-compliant balloon with microsurgical blades; intimal up to medial incisions allow deeper plaque fracture	Dual-layer balloon design; inflations up to 35–40 atm; pure mechanical compression/fracture
Optimal lesion morphology	Eccentric or concentric calcium	Eccentric or concentric calcium; fibrotic tissue, fibro-calcific ISR; resistant eccentric lesions	Eccentric or concentric calcium; stent underexpansion
Sizing & inflation strategy	Size ∼1:1 to vessel; gradual stepwise inflations (12–20 atm)	Size ∼1:1 (slight undersize if tortuous); inflate at 6–12 atm; when undersized and higher inflation pressure can be applied; Multiple inflations are recommended.	Undersize ∼0.6–0.7 × RVD; slow, step-up inflations; use buddy wire to prevent wire loss
Strengths	Good deliverability; controlled plaque modification; safe	Predictable plaque modification; effective at low pressures; improves stent expansion; useful in ISR, potential dose dependent effect	Unmatched dilating force; highly effective in undilatable lesions; might allows avoiding atherectomy if crossable
Limitations/pitfalls	Limited efficacy in deep or nodular calcium; possible need for escalation	Reduced deliverability; risk of blade fracture; perforation if oversized/aggressive	Reduced trackability and deliverability; risk of wire entrapment; not first-line
Key evidence	AngioSculpt, NaviScore, Aperta NSE studies; Wedge NC RCT; PREPARE-CALC; RCT meta-analysis	Cutting Balloon Global RCT, RESCUT, COPS trial, ROTA-CUT strategies; RODIN-CUT case series publication; SHORT-CUT trial	ISAR-CALC RCT (↑MLD vs scoring); PREPARE-CALC; OCT registries; VICTORY Trial

Intravascular imaging is central to the success of all these strategies. Angiography alone lacks the ability to fully characterize calcium morphology. By contrast, IVUS, and particularly OCT with AI-powered software, allow quantification of circumferential extent, depth, and thickness of calcium, enabling selection of the most appropriate device. Imaging also permits direct confirmation of calcium fracture and adequate lesion preparation before stenting. The experience of the RODIN-CUT technique illustrates how imaging can generate novel procedural insights and improve the performance of existing tools.

Looking forward, further evidence from randomized studies is needed to better define the long-term outcomes of balloon-based calcium modification compared with atherectomy or IVL. Head-to-head data comparing cutting vs. scoring balloons are still lacking, and the biological implications of dose-dependent cutting balloon inflations remain to be validated. Future device innovation may focus on hybrid balloons that combine cutting or scoring elements with ultra-high-pressure capabilities, potentially bridging current gaps in the treatment of calcified coronary disease.

## Conclusion

Dedicated calcium-modifying balloons—scoring, cutting, and OPN NC™—are key tools for the preparation of balloon-crossable calcified lesions. Guided by intravascular imaging, they enable safe and predictable plaque modification, improving stent expansion and long-term outcomes. Emerging strategies such as the RODIN-CUT technique highlight that optimizing the use of existing devices can further enhance efficacy. In practice, these balloons should be viewed as complementary within a multimodality approach that includes IVL or atherectomy when necessary. Ongoing trials will help refine their precise role in the management of complex calcified coronary artery disease.
